# Impact of high-sensitivity troponin elevation and dynamic changes on 90-day mortality in patients with acute ischemic stroke after mechanical thrombectomy: results from an observational cohort

**DOI:** 10.1136/jnis-2022-019682

**Published:** 2022-12-12

**Authors:** Feng Chen, Xiaolin Bai, Xiuzhe Wang, Liren Zhang, Feng Wang, Ligang Huang, Jiangshan Deng, Zhi Geng

**Affiliations:** Department of Neurology, Shanghai Sixth People's Hospital Affiliated to Shanghai Jiao Tong University School of Medicine, Shanghai, China

**Keywords:** Thrombectomy, Stroke

## Abstract

**Background:**

A study was undertaken to evaluate the impact of high-sensitivity cardiac troponin I (hs-cTnI) elevation and hs-cTnI dynamic changes on 90-day mortality in patients with acute ischemic stroke (AIS) treated with mechanical thrombectomy (MT).

**Methods:**

Patients with AIS receiving MT were included in the study. Sixty hours after AIS onset, hs-cTnI levels were measured before and after MT to determine elevated and dynamic changes. Patients were stratified into either normal or hs-cTnI elevation groups according to the pre-MT hs-cTnI cut-off value of 0.03 ng/L. hs-cTnI dynamic changes were defined as an increase or decrease of more than 20% pre-MT and post-MT, and at least one hs-cTnI level >0.03 ng/L. Multivariate Cox regression models were used to investigate the association between hs-cTnI elevation, hs-cTnI dynamic changes, and 90-day mortality in patients with AIS after MT.

**Results:**

A total of 423 patients with AIS after MT were included in our final analysis, of whom only 72 (17%) showed hs-cTnI elevation. Post-MT hs-cTnI retesting was performed in 354 patients, and 90 (25.4%) patients presented with hs-cTnI dynamic changes. 119 patients died within 90 days. After adjusting for potential confounding factors, the Cox regression model showed that patients with hs-cTnI dynamic changes, rather than hs-cTnI elevation, were associated with 90-day mortality (p<0.05). Compared with the hs-cTnI non-dynamic changes, these results showed that a statistical association was present between rising hs-cTnI dynamic changes and 90-day mortality (p>0.05).

**Conclusions:**

hs-cTnI dynamic changes, dominated by the rising pattern rather than hs-cTnI elevation, were independent factors associated with 90-day mortality in patients with AIS after MT, especially in elderly subjects.

**WHAT IS ALREADY KNOWN ON THIS TOPIC**

- Elevated cardiac troponin (cTn) levels are associated with mortality in patients with acute ischemia (AIS). cTnI and cTnT performed differently in mortality prediction. The role of hs-cTnI or hs-cTnI dynamic changes in the mortality of patients with AIS after mechanical thrombectomy (MT) in 90 days is unclear and rarely investigated.

**WHAT THIS STUDY ADDS**

- Dynamic changes in high-sensitivity cardiac troponin I (hs-cTnI) rather than hs-cTnI elevation are an independent factor associated with mortality of patients with AIS after MT in 90 days.

**HOW THIS STUDY MIGHT AFFECT RESEARCH, PRACTICE OR POLICY**

- Even if the hs-cTnI test before MT was negative, the difference between hs-cTnI retesting of more than 20% could still predict an unfavorable event in patients with AIS. It is therefore necessary for patients with AIS who are candidates for MT to take consecutive cTn tests before and after MT, especially those who are elderly.

## Intrduction

Based on the results from multiple randomized controlled trials, mechanical thrombectomy (MT) is considered the best treatment strategy for specific patients with acute ischemic stroke (AIS) with large vessel occlusion (LVO), which substantially reduces disability.^1^ However, the 90-day mortality is relatively high at 28% in patients with AIS with successful recanalization.^2^ In addition to improving the recanalization rate through technical progress, there is an urgent need to find strategies to reduce mortality in patients with AIS after MT.

Cardiac complications are common in patients with AIS. In patients with first-ever AIS onset and no previous comorbid cardiovascular conditions, the risk of major adverse cardiovascular events within 30 days was 25 times higher than in individuals without AIS.^3^ Cardiac troponin (cTn), an essential component of the contractile apparatus within cardiac myocytes, is considered the gold standard biomarker of myocardial injury.^4^ The high-sensitivity cardiac troponin (hs-cTn) assay allows detection at a 10–100-fold lower cut-off value than conventional methods,^5^ which significantly improves the early diagnostic accuracy of acute myocardial infarction (AMI). According to the previous universal definition of AMI, cTn elevation above the 99th percentile of a normal reference population—namely, upper reference limit (URL) with a coefficient of variation <10%—was a mandatory feature.^6^ However, other clinical conditions such as congestive heart failure, ventricular hypertrophy, and chronic kidney disease could also cause chronic cTn release,^7^ which weakens the accuracy of cTn in AMI diagnosis. Thus, with the widespread use of hs-cTn, acute myocardial injury is defined as an increase or decrease in cTn values (>20%) with at least one value above the 99th percentile URL.^4^ Although cTn isotypes (cTnI and cTnT) are interchangeable in diagnosing AMI, they performed differently in mortality prediction.^8 9^ cTnT may have superiority in predicting non-cardiovascular death, while cTnI tends to be more efficient for some cardiovascular disease outcomes.^10^

Evidence has shown that elevated cTn is associated with mortality in patients with AIS in the long or short term.^11 12^ However, the distribution of hs-cTnI elevation or hs-cTnI dynamic changes in patients who underwent MT and its role in the 90-day mortality is unclear and is rarely investigated. We therefore studied the prognostic association of hs-cTnI elevation and hs-cTnI dynamic changes in a cohort of consecutive patients receiving MT.

## Methods

### Study design and setting

This was a single-center observational cohort study conducted at the Shanghai Sixth People’s Hospital affiliated to Shanghai Jiao Tong University School of Medicine. All consecutive patients who received emergency MT for AIS within 24 hours of symptom onset were eligible for inclusion. The following patients were excluded: (1) patients whose hs-cTnI levels were not measured on admission; (2) patients with cerebral hemorrhage as the primary disease comorbid with cerebral infarction; and (3) patients aged <18 years.

In our center, patients with suspected neurological deficits within 24 hours of symptom onset are rapidly estimated by a well trained team available 24 hours per day. As a tertiary hospital, patients from nearby hospitals where conditions do not permit them to conduct MT are transferred to our center.

Patients with LVO who fit the treatment criteria receive emergency MT therapy by interventional radiologists. From February 2016 to November 2020, the indications for emergency MT were National Institutes of Health Stroke Scale (NIHSS) score ≥6, Alberta Stroke Program Early CT Score (ASPECTS)^13^ ≥6, anterior circulation occlusion on CT angiography (CTA) or magnetic resonance angiography (MRA), and time of admission within 6 hours of symptom onset. After introducing multi-model CT devices in December 2020, the time window for MT was expanded from 6 to 24 hours from stroke onset if patients with anterior LVO met the DAWN or DEFUSE-3 criteria in CT perfusion imaging.^14 15^ In addition, patients receiving MT with posterior circulation LVO within 24 hours of stroke onset were also included. In our center, a Solitaire stent (ev3 Neurovascular, Irvine, California, USA) with an intracranial support catheter for MT is the preferred treatment. A maximum of three passes are allowed for retrieval attempts in every target artery. Balloon dilation or stent implantation is considered a remedial measure if large artery atherosclerosis stenosis is identified or re-occlusion occurs during MT. After MT, all patients are administered appropriate antithrombotic and lipid-lowering therapies according to the guidelines.^16^

To detect a difference in pre-MT cTn elevation of 90-day mortality, which is in agreement with the study by Yi *et al* 17 with a two-sided 5% significance level and a power of 90%, a sample size of 68 patients per group was needed given an anticipated dropout rate of 15%. We planned to recruit 408 patients (1:5 allocation to cTn elevation and control groups).

### Data collection and outcomes

All data were collected immediately as the first value in the initial 7 days following admission. The following variables were collected: (1) basic demographics including age, sex, weight, and height; (2) vascular risk factors including hypertension, diabetes, and smoking; (3) comorbidities including atrial fibrillation (AF), coronary artery disease (CAD), and previous stroke; (4) treatment status including previous antithrombotic therapy (antiplatelet or anticoagulation therapy), onset-to-admission time (OTA), transfer from the referring hospital, use of intravenous recombinant tissue plasminogen activator (IV rtPA) and dual antiplatelet therapy after MT according to the guidelines,^16^ and modified Thrombolysis in Cerebral Infarction (mTICI) score. Stroke severity was assessed using the NIHSS score. Poor reperfusion was defined as a mTICI score of 0–2 a; (5) laboratory tests including the first hs-cTnI test on admission (pre-MT hs-cTnI), hs-cTnI retesting at 06:00 hours the following day (post-MT hs-cTnI), baseline blood glucose, glycated hemoglobin (HbA1c), homocysteine (HCY), platelet count, international normalized ratio (INR), low-density lipoprotein (LDL-C), and creatinine; and (6) stroke location including posterior circulation involvement and insula involvement, and hemorrhage transformation identified by CT or MRI. The hs-cTnI levels were detected using microparticle enzymatic luminescence (UniCel Dxi 800, Beckman, USA). For this assay, the lower detection limit was 0.001 μg/L and the 99th percentile of the URL was 0.03 μg/L. Elevation of hs-cTnI was defined as pre-MT hs-cTnI level >0.03 μg/L. A rise or fall of more than 20% in pre-MT and post-MT hs-cTnI levels and at least one hs-cTnI value above the URL on serial measurements were considered hs-cTnI dynamic changes according to the Fourth Universal Definition of Myocardial Infarction.^4^

The estimated glomerular filtration rate (eGFR) was calculated using the Cockcroft–Gault formula. Patients were followed up for 90 days from the first day of admission. All patients underwent routine cranial CT examination within 24–36 hours after MT if their condition permitted. Intracranial hemorrhage (ICH) was defined as bleeding observed on the follow-up CT. Symptomatic ICH (sICH) was defined as ICH with an increase in NIHSS score of ≥2 points at 36 hours after MT,^18^ and survival status was collected on day 90 by telephone inquiry. For patients who were lost to follow-up on day 90, the survival status and last survival time were acquired by referring to the latest electronic medical records.

### Management of missing data

As this was an observational study, missing data were inevitable. More than 15% of the data regarding HCY and HbA1c were missing, and these parameters were not qualified for the following analysis. For variables with missing data in fewer than 15% of patients, the missing data were filled with predictors using multiple imputations to minimize the bias resulting from missing values. Detailed information on the missing data are shown in online supplemental figure 1.

10.1136/jnis-2022-019682.supp1 Supplementary data

### Statistical analysis

Normally distributed continuous variables were presented as mean±SD, skewed continuous variables as median (IQR), and categorical variables as absolute values (%). Differences between continuous variables in the two groups were analyzed by t-test or Mann–Whitney U test as appropriate. Differences between categorical variables in two or more groups were analyzed using Pearson’s χ^2^ test or Fisher’s exact probability test as appropriate. Univariate analysis was performed using the Kaplan–Meier survival test to analyze the survival curves for each variable. The cumulative risk function was used to test whether the data met the proportional hazards hypothesis. After testing the collinearity and proportional hazards hypothesis, variables with p<0.1 in the univariate analysis were included in the multiple Cox regression (Forward: LR method). The performance of the variant in predicting survival status was assessed using receiver operating characteristic (ROC) curves. A subgroup analysis was performed to further investigate the association between hs-cTnI levels and mortality after MT in different patients. All statistical tests were two-tailed and statistical significance was set at p<0.05. All analyses were performed using SPSS version 25.0 (IBM, Armonk, New York, USA) and Medcalc software 20.0.22 (MedCalc Software, Ostend, Belgium). Figures were drawn using Medcalc software 20.0.22, Excel 2016, and Point software 2016 (Microsoft, Redmond, Washington, USA).

## Results

### Characteristics of the study subjects

Between February 2016 and May 2022, 518 patients were treated with emergency MT at our hospital. Based on the protocol (see online supplemental figure 2), two patients were excluded because the primary disease was cerebral hemorrhage. Ninety-three patients had no pre-MT hs-cTnI measurements. The excluded patients had shorter OTA, higher proportions of transfer, and diabetes mellitus (p<0.05; see online supplemental table 1). Therefore, 423 patients were finally enrolled in the study, comprising 248 men (58.6%) and 175 women (41.4%), with a mean age of 73±12 years. Of the 423 patients, 354 patients underwent post-MT hs-cTnI measurements. Pre-MT hs-cTnI and post-MT hs-cTnI sampling were 1.77 (IQR 1.00–4.00, minimum 0, maximum 23.97) and 20.00 hours (IQR 14.00–23.75, minimum 4.00, maximum 54.00) from onset, respectively. Patients with serial hs-cTnI measurements had a higher proportion of previous stroke (p<0.05; see online supplemental table 2).

In this study, 72 of the 423 patients (17.0%) had hs-cTnI elevation. Ninety (25.4%) of the 354 patients who underwent post-MT measurements had hs-cTnI dynamic changes, with a rising pattern in 75 and a falling pattern in 15 patients. Of the 423 patients, three (0.7%) were diagnosed with AMI, of which one survived and suffered AIS during percutaneous transluminal coronary intervention for AMI. The other two patients with AMI died on day 3 and day 17 after AIS admission, respectively. Of the 423 patients, 19 (4.5%) had no CT or MRI examinations because of severe conditions. sICH occurred in 110/404 (27.2%) patients. Of the remaining 404 patients, 72 (17.8%) had insula involvement. A higher proportion of patients without insula involvement had hypertension (online supplemental table 3). During the 3-month follow-up period, 35/423 (8.3%) patients were lost to follow-up and 119 of the remaining 388 (30.7%) patients died. There was no significant difference in the mortality between February 2016 and November 2020 (61/216, 28.2%) and between December 2020 and May 2022 (58/172, 33.7%; p>0.05).

### Univariable analysis for characteristics of elevated hs-cTnI and hs-cTnI dynamic changes

In the hs-cTnI elevation group, patients had more comorbid AF and CAD, more previous antithrombotic therapy, higher hs-cTnI dynamic change rate, higher INR, more insula involvement, a lower level of LDL-C, and higher mortality than the comparison group (all p<0.05; see online supplemental table 4). Compared with patients without hs-cTnI dynamic changes, the hs-cTnI dynamic changes group tended to have a greater proportion of elderly and female patients, more comorbid AF and CAD, more previous antithrombotic therapy, higher NIHSS score on admission, less dual antiplatelet therapy, lower platelet levels, higher INR, lower eGFR, and higher mortality (all p<0.05, table 1). The hs-cTnI elevation on admission and hs-cTnI dynamic changes were not associated with ICH or sICH (all p>0.05).

**Table 1:** Clinical characteristics and mortality according to troponin dynamic changes

	hs-cTnI dynamic changes		P value
	Yes (n=90)	No (n=264)	
Age >70 years, n (%)	65 (72.2)	138 (52.3)	**0.001**
Male, n (%)	44 (48.9)	163 (61.7)	**0.033**
Vascular risk factors			
Hypertension, n (%)	62 (68.9)	168 (63.6)	0.367
Diabetes mellitus, n (%)	24 (26.7)	74 (28.0)	0.803
Current smoking, n (%)	11 (12.2)	47 (17.8)	0.217
Comorbidities			
AF, n (%)	56 (62.2)	123 (46.6)	**0.010**
CAD, n (%)	15 (16.7)	24 (9.1)	**0.047**
Previous stroke, n (%)	19 (21.1)	43 (16.3)	0.299
Treatment status			
Previous antithrombotic therapy, n (%)	22 (24.4)	40 (15.2)	**0.045**
OTA, min (IQR)	89 (57–224)	107 (60–240)	0.549
Transfers, n (%)	9 (10)	39 (14.8)	0.253
Use of IV rtPA, n (%)	21 (23.3)	73 (27.7)	0.423
Poor reperfusion, n (%)	22 (24.4)	43 (16.3)	0.084
Dual antiplatelet therapy, n (%)	5 (5.6)	36 (13.6)	**0.039**
NIHSS on admission			**0.001**
0–8*, n (%)	3 (3.3)	42 (15.9)	
9–16, n (%)	29 (32.2)	103 (39.0)	0.021†
>16, n (%)	58 (64.4)	119 (45.1)	**<0.001**†
Laboratory tests			
Baseline blood glucose, mmol/L (IQR)	7.15 (6.30–8.50)	7.10 (6.10–8.80)	0.921
HbA1c,% (IQR)	6.00 (5.50–6.50)	5.90 (5.50–7.00)	0.754
HCY, μmol/L (IQR)	15.43 (11.41–20.05)	13.25 (9.91–18.20)	0.068
Platelets, 10^9^/L (IQR)	176 (150–219)	199 (160–235)	**0.036**
INR (IQR)	1.04 (0.98–1.12)	1.02 (0.94–1.09)	**0.022**
LDL-C, mmol/L (IQR)	2.32 (1.83–3.16)	2.63 (1.95–3.39)	0.083
eGFR, L/min/1.73 m^2^ (IQR)	53.81 (42.04–75.90)	66.32 (48.59–80.99)	**0.002**
Stroke location			
Posterior circulation, n (%)	33 (36.7)	84 (31.8)	0.398
Insula‡, n (%)	12 (14.1)	51 (19.6)	0.255
ICH‡	25 (29.4)	86 (33.1)	**0.530**
sICH‡	22 (25.9)	69 (26.5)	**0.905**
Mortality§, n (%)	37 (45.7)	57 (23.5)	**<0.001**

*Control group.

†P<0.017 was statistically significant.

‡Data were available in 345 patients.

§Data were available in 324 patients.

AF, atrial fibrillation; CAD, coronary artery disease; eGFR, estimated glomerular filtration rate; HbA1c, glycated hemoglobin; HCY, homocysteine; hs-cTnI, high-sensitivity cardiac troponin; ICH, intracranial hemorrhage; INR, international normalized ratio; IQR, interquartile range; LDL-C, low-density lipoprotein; NIHSS, National Institutes of Health Stroke Scale score; OTA, onset-to-admission time; IV rtPA, intravenous recombinant tissue plasminogen activator; sICH, symptomatic intracranial hemorrhage.

### Analysis for predictors of mortality in 90 days

**Figure 1:**
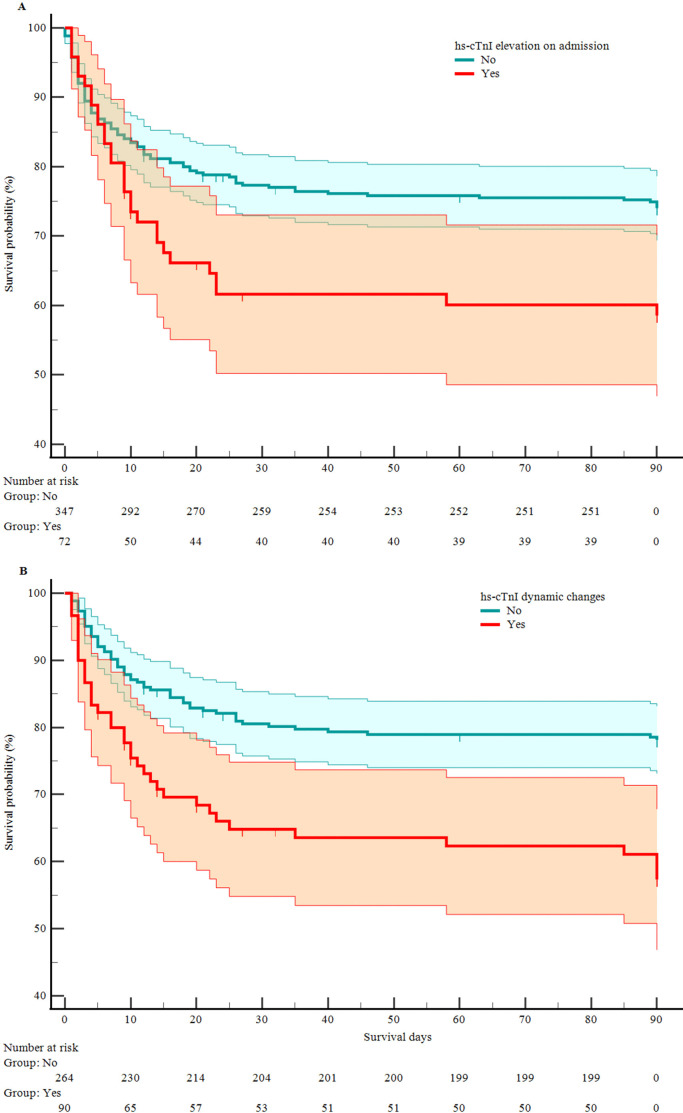
Kaplan–Meier curves of effect of high-sensitivity cardiac troponin I (hs-cTnI) elevation on admission (A) and hs-cTnI dynamic changes (B) on survival. Shaded areas represent 95% CI.

Continuous variables were divided into categorical variables by the values with clinical significance, given the clinical utility of the model. The Kaplan–Meier test indicated that hs-cTnI elevation on admission (HR 1.907, 95% CI 1.162 to 3.131, p<0.05) and hs-cTnI dynamic changes (HR 2.618, 95% CI 1.610 to 4.256, p<0.05) were associated with higher mortality. Survival plots are shown in figure 1. Model 1 was adjusted for age >70 years, hypertension, use of IV rtPA, poor reperfusion, double antiplatelet therapy, NIHSS score on admission, baseline blood glucose >7 mmol/L, eGFR <60 L/min/1.73 m^2^, and posterior circulation. In addition to the mortality covariates in Model 1, sICH was added to Model 2. Multiple Cox regression analysis showed that the hs-cTnI dynamic changes were independently associated with 90-day mortality of patients with AIS after MT (table 2; the complete analysis is shown in online supplemental table 5). Comparisons of multiple Cox regression results between the original data and multiple imputations are shown in online supplemental tables 6-7. This association persisted after excluding three patients with AMI (Model 1: HR 1.601, 95% CI 1.043 to 2.457, p<0.05; Model 2: HR 1.659, 95% CI 1.029 to 2.673, p<0.05). Online supplemental figure 3 shows that, compared with patients without dynamic changes, patients with hs-cTnI rising dynamic changes had a higher proportion of mortality (χ^2^=9.978, p=0.002). However, hs-cTnI falling dynamic changes were not associated with mortality (p>0.05). There was no significant difference in mortality between an increase and decrease in hs-cTnI (p>0.05).

**Table 2:** Kaplan–Meier survival analysis and multivariate Cox regression analysis of the predictors of mortality

	Univariate model		Model 1		Model 2	
	HR (95% CI)	P value	aHR (95% CI)	P value	aHR (95% CI)	P value
hs-cTnI elevation on admission	1.907 (1.162 to 3.131)	0.011	–	>0.05	–	>0.05
hs-cTnI dynamic changes	2.618 (1.610 to 4.256)	<0.001	1.613 (1.056 to 2.463)	0.027	1.597 (1.022 to 2.495)	0.040

Model 1: adjusted for age >70, hypertension, use of IV recombinant tissue plasminogen activator, dual antiplatelet therapy, poor reperfusion, National Institutes of Health Stroke Scale score on admission, baseline blood glucose >7 mmol/L, estimated glomerular filtration rate <60 L/min/1.73 m^2^, and posterior circulation.

Model 2: adjusted for age >70, hypertension, use of IV recombinant tissue plasminogen activator, dual antiplatelet therapy, poor reperfusion, National Institutes of Health Stroke Scale score on admission, baseline blood glucose >7 mmol/L, estimated glomerular filtration rate <60 L/min/1.73 m^2^, posterior circulation, and symptomatic intracranial hemorrhage.

hs-cTnI, high-sensitivity cardiac troponin.

### ROC analysis

**Figure 2:**
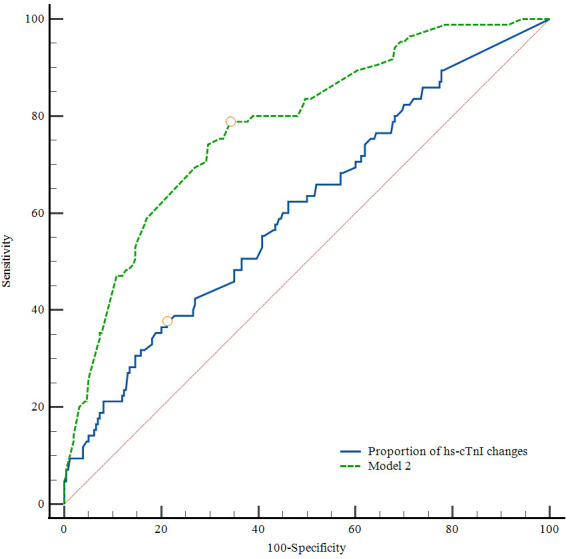
ROC curves of proportions of high-sensitivity cardiac troponin I (hs-cTnI) changes and Model 2 for the prediction of mortality in 90 days. hs-cTnI dynamic change (area under the curve (AUC) 0.612; cut-off value 165.93%; Youden index J, 0.173; sensitivity 36.17%; specificity 81.15%, p<0.05). Model 2 (AUC 0.778; cut-off value 0.212; Youden index J, 0.446; sensitivity 78.8%; specificity 65.8%, p<0.05).

ROC curve analysis showed that the proportion of hs-cTnI changes was the parameter with the highest area under the curve (AUC 0.612) for the prediction of mortality of patients with AIS after MT in 90 days, with a cut-off point of 165.93% for maximum sensitivity of 36.17% and specificity of 81.15%. The AUC of Model 2 was 0.778, with a cut-off point of 0.212 for maximum sensitivity of 78.8% and specificity of 65.8%. (figure 2).

### Subgroup analysis

Subgroup analysis showed that hs-cTnI dynamic changes were associated with mortality in female patients, patients without AF, patients without insula involvement, those with anterior circulation involvement, and patients without sICH. hs-cTnI dynamic changes interacted with age (online supplemental table 8).

## Discussion

In this single-center cohort of 423 consecutive AIS patients with LVO who received MT, we found associations between elevated pre-MT hs-cTnI levels and hs-cTnI dynamic changes before and after MT with 90-day survival. We observed that pre- and post-MT hs-cTnI dynamic changes, especially rising changes, rather than pre-MT elevated hs-cTnT levels were independent predictors of 90-day survival when adjusted for covariates. However, we did not observe an association between pre-MT elevated hs-cTnT levels and pre- and post-MT hs-cTnI dynamic changes with sICH.

Our primary exclusion criteria were patients transferred from other hospitals by ambulance as those who were candidates for MT always had taken necessary laboratory tests (some of them without hs-cTnI tests) before admission to our center. To save time, blood sampling was often omitted. In additon, the heterogeneous interhospital testing methods and cut-off values made the analysis difficult.

Many published studies have shown that cTn elevation is a risk factor for mortality in patients with AIS, even in those who underwent thrombolysis or MT therapy.^17 19 20^ The rate of abnormal cTn levels in patients with AIS is approximately 9.7–60%, depending on the various testing methods and cut-off value.^11^ Patients with elevated cTn tended to be female, older, and had more cardiac comorbidities and renal impairment, which is similar to our results. However, in our study, although pre-MT hsTnI was a strong predictor of mortality in univariate analysis, the marker did not provide additional prognostic information besides hs-cTnI dynamic changes before and after MT. These results were similar to those of previous studies.^21 22^ One possible explanation is the sensitivity of hs-cTnI. Compared with the conventional cTn test, the relatively low cut-off value of hs-cTnI could put more ‘normal’ patients in the positive group, which may hardly result in a significant difference between the two groups. Furthermore, the time between the initiation of symptoms and the first cTn test could have caused heterogeneous results in different studies. A previous study showed that the time to cTnI assessment was independently associated with the diagnostic accuracy of AMI. The initial cTnI testing taken 4.5 hours after the occurrence of symptoms could more accurately help to identify myocardial damage.^23^

Several studies have shown that dynamic cTn changes are associated with cardiac or non-cardiac death. Even minor changes in cTn levels had prognostic implications.^24 25^ Our study showed that hs-cTnI dynamic change was independently associated with the 90-day mortality of patients with AIS after MT, further verifying the prognostic value of hs-cTnI dynamic changes, which was rarely explored in previous MT-related studies.

The etiology of hs-cTnI dynamic changes in predicting the 90-day mortality in patients with AIS after MT remains unclear. Stroke-heart syndrome, which involves dysregulation of the central autonomic network, is the most recognized mechanism of myocardial injury in patients with AIS. The stress induced by the occurrence of stroke could lead to functional and structural alterations in the heart, similar to catecholamine toxicity, triggering diseases such as arrhythmia, Takotsubo syndrome, and even coronary demand ischemia (type 2 myocardial infarction). Insular infarct has been shown to be associated with dynamic hs-cTnT changes,^26^ which may be because of the role of the insula in cardiovascular autonomic function and dysregulation after infarction. However, in the subgroup analysis in our study, dynamic hs-cTnI changes were associated with mortality in patients without insula injury. There is little literature on the association between hs-cTnI levels and insula injury. We can only speculate about the possible causes of this observation by the different performances of cTnI and cTnT in death from non-cardiovascular and cardiovascular disease. Both cTnI and cTnT were risk factors for all-cause mortality, but cTnT rather than cTnI was an independent risk factor for non-cardiovascular disease.^9^ Although the source of both cTnI and cTnT is mainly cardiac injury, the level of cTnI seems prone to be influenced by cardiac etiology. In our study, patients without insula involvement may have had more cardiac mortality due to the higher proportion of hypertension, which could explain the difference in insula subgroups.

We also observed that patients in the hs-cTnI dynamic changes group tended to have a higher INR and low platelet count, despite the lower application of double antiplatelet therapy. One possible explanation is that patients in the hs-cTnI dynamic changes group had more cardiac comorbidities than those in the comparison group. Thus, they received more antithrombotic therapy before admission. A study showed that cTnI dynamic changes had a more robust statistical association with hemorrhagic transformation after thrombolysis in patients with AIS than troponin elevation.^27^ However, in patients with AIS after MT, we did not observe an association between troponin elevation and troponin dynamic changes with sICH and a study of hs-cTnT showed similar results.^20^ Further studies on the mechanism of hs-cTnI dynamic change in predicting the 90-day mortality in patients with AIS after MT are warranted.

In clinical practice, there is a dilemma in interpreting and managing cTn elevation in patients with AIS. Although guidelines recommend the need for cTn tests in patients with AIS, there is no clear advice on the treatment of patients with elevated cTn.^16^ However, some researchers claim that cTn should not be included in routine detection, as they found that cTn elevation rarely changed the immediate clinical management in hospitalized patients with AIS.^28^ Furthermore, it is challenging for clinicians to distinguish whether myocardial injury is induced by stroke-heart syndrome or AMI. A study showed that cTnI levels >2 μg/L should not be attributed to AIS but should be investigated further for coronary factors.^29^ Our study showed that the proportion of hs-cTnI changes >165.93% was highly specific in predicting the death of patients with AIS after MT but had low sensitivity. Furthermore, the sensitivity of Model 2 was relatively high. In clinical practice, Model 2 can be used as a tool for preliminary screening, and the proportion of hs-cTnI changes enables further confirmation in high-risk target patients. Even if the pre-MT hs-cTnI test was negative, a difference between pre-MT hs-cTnI and post-MT hs-cTnI of more than 20% could still predict the unfavorable events in patients with AIS. Therefore, it is necessary for patients with AIS who are candidates for MT to test cTn before and after MT, especially elderly patients. Early detection and treatment of myocardial injury could improve the survival rate of patients with AIS after MT. Large-scale prospective studies and clinical trials are warranted to explore cut-off values for cTn dynamic changes in patients with AIS, confirm this association, and to test whether patients would benefit from early intervention.

This study has some limitations. First, it is a single-center, observational, moderate-sample study which limits generalizability. Our results still require further confirmation, especially since the association between pre-MT hs-cTnI elevation and mortality was insignificant after adjustment. Second, we adopted composite death as the endpoint rather than the specific cause of death for each patient. Even if the recanalization was successful, some patients died after MT without imaging tests, making the actual causes of death (such as hemorrhagic transformation, encephaledema, or reocclusion) challenging to investigate. Third, the diagnosis of AMI mainly relied on symptoms, dynamic electrocardiography, and troponin changes. Some patients with AMI with an altered mental status could not identify symptoms and could thus be missed without careful investigation. Fourth, some meaningful missing data were missing in our study, although we attempted to use multiple imputations to compensate. Fifth, as the clinical data were collected by referring to electronic medical records, there was a lack of clearly standardized collection times for hs-cTn. Finally, this study was subject to referral bias as it was performed at a comprehensive stroke center.

## Conclusion

In this Chinese single-center cohort study of patients with AIS requiring MT, pre- and post-MT hs-cTnI dynamic changes comprising both rising and falling patterns rather than pre-MT hs-cTnI elevation were independent factors associated with 90-day mortality, especially in elderly patients. Compared with patients with non-dynamic hs-cTnI changes, those with rising hs-cTnI dynamic changes were prone to have a higher rate of mortality in 90 days. However, there were no associations between pre-MT elevated hs-cTnT and pre- and post-MT hs-cTnI dynamic changes with sICH. Further studies are required to confirm these results.

10.1136/jnis-2022-019682.supp2 Supplementary data

## Data Availability

Data are available upon reasonable request.
