# Supplementary material for: Impact of high-sensitivity troponin elevation and dynamic changes on 90-day mortality in patients with acute ischemic stroke after mechanical thrombectomy: results from an observational cohort
Source: J Neurointerv Surg. 2022 Dec 12;15(11):1142–7. doi: 10.1136/jnis-2022-019682 (PMC10579475; doi:10.1136/jnis-2022-019682)
Supplement: Supplementary data [file jnis-2022-019682supp001.pdf]

Figure S1. Summary of missing data.

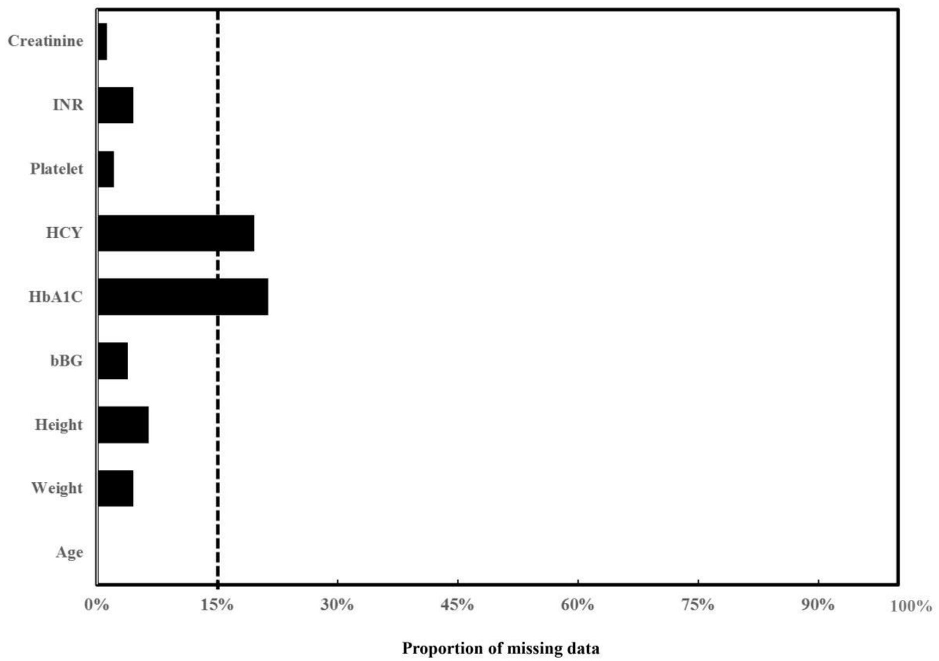

Black bars indicate variables with missing data for more than 15% of patients. INR, international normalized ratio; HCY, homocysteine; HbA1C, glycated hemoglobin; bBG, baseline blood glucose.

Figure S2. Protocol flow chart in this study.

BMJ Publishing Group Limited (BMJ) disclaims all liability and responsibility arising from any reliance placed on this supplemental material which has been supplied by the author(s)

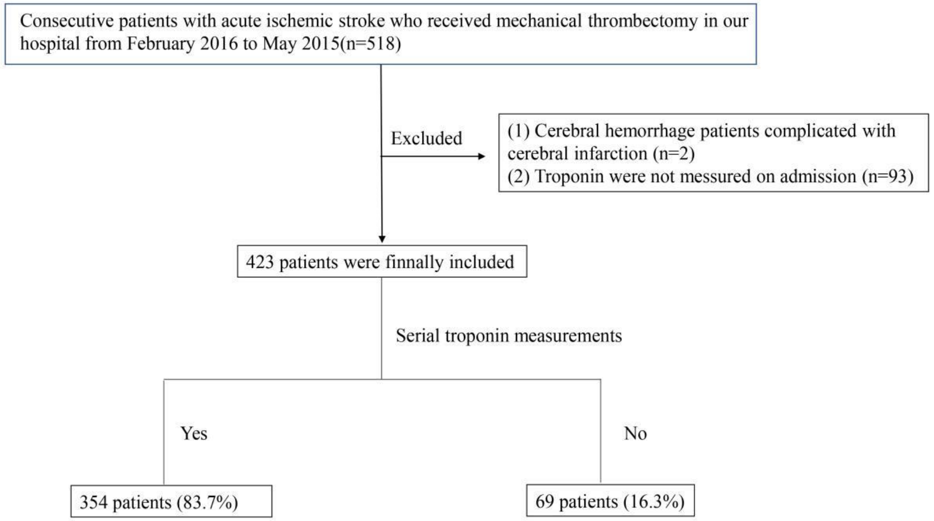

Table S1 Clinical characteristics and mortality of patients, stratified by the presence of hs-cTnI measurement before MT

	hs-cTnI measurement before MT		*P*
	Yes (n=423)	No (n=95)	
Age >70, n (%)	246(58.2)	46(48.4)	0.084
Male, n (%)	248(58.6)	58(61.1)	0.664
**Vascular risk factors**			

Hypertension, n(%)	275(65.0)	60(63.2)	0.733
Diabetes mellitus, n(%)	116(27.4)	14(14.7)	**0.010**
Current smoking, n(%)	75(17.7)	18(18.9)	0.780
**Comorbidities**			
AF, n(%)	212(50.1)	44(46.3)	0.503
CAD, n(%)	42(9.9)	7(7.4)	0.441
Previous stroke, n(%)	66(15.6)	16(16.8)	0.765
**Treatment status**			
OTA, min (IQR)	106(60-240)	215(78-335)	**0.001**
Transfers, n(%)	56(13.2)	52(54.7)	**<0.001**
Use of IV rtPA, n(%)	115(27.2)	21(22.1)	0.309
Poor reperfusion, n(%)	84(19.9)	22(23.2)	0.471
**NIHSS on admission**			
0-8*, n(%)	59(13.9)	9(9.5)	0.411
9-16, n(%)	149(35.2)	32(33.7)	
>16, n(%)	215(50.8)	54(56.8)	
**Mortality†, n%**	119(30.7)	18(20.7)	0.063

*control group

†data were available from 388 patients in the "Yes" group and 87 patients in the "No" group considering the patients lost to follow-up.

hs-cTnI, high-sensitivity cardiac troponin; MT, mechanical thrombectomy; AF, atrial fibrillation; CAD, coronary artery disease; OTA, onset-to-admission time; IQR, interquartile range; IV rtPA, intravenous recombinant tissue plasminogen activator; NIHSS, National Institutes of Health Stroke Scale score.

Table S2. Clinical characteristics and mortality of patients, stratified by the presence of serial measurements in hs-cTnI

	Serial measurements in hs-cTnI		*P*
	Yes (n=354)	No (n=69)	
Age >70, n (%)	203(57.3)	43(62.3)	0.444

Male, n (%)	207(58.5)	41(59.4)	0.884
**Vascular risk factors**			
Hypertension, n(%)	230(65.0)	45(65.2)	0.969
Diabetes mellitus, n(%)	98(27.7)	18(26.1)	0.786
Current smoking, n(%)	58(16.4)	17(24.6)	0.101
**Comorbidities**			
AF, n(%)	179(50.6)	33(47.8)	0.677
CAD, n(%)	39(11.0)	3(4.3)	0.090
Previous stroke, n(%)	62(17.5)	4(5.8)	**0.014**
**Treatment status**			
Previous antithrombotic therapy, n%	62(17.5)	10(14.5)	0.541
OTA, min (IQR)	105(60-240)	120(59-278)	0.531
Transfers, n(%)	48(13.6)	8(11.6)	0.659
Use of IV rtPA, n(%)	94(26.6)	21(30.4)	0.507
Poor reperfusion, n(%)	65(18.4)	19(27.5)	0.081
Dual antiplatelet therapy	4(5.6)	46(13.1)	0.071
**NIHSS on admission**			
0-8*, n(%)	45(12.7)	14(20.3)	0.071
9-16, n(%)	132(37.3)	17(24.6)	
>16, n(%)	177(50.0)	38(55.1)	
**Laboratory test**			
hs-cTnI elevation on admission, n%	62(17.5)	10(14.5)	0.541
Baseline blood glucose, mmol/L (IQR)	7.10(6.10-8.70)	6.80(5.90-8.60)	0.379
HbA1c,% (IQR)	5.90(5.50-6.83)	6.00(5.60-7.70)	0.292
HCY, µmol/L (IQR)	13.60(10.28-18.90)	12.88(10.05-16.93)	0.279

Platelet, 10^9/L (IQR)	193(155-230)	184(154-235)	0.843
INR (IQR)	1.02(0.95-1.09)	1.04(0.96-1.10)	0.300
LDL-C mmol/L (IQR)	2.53(1.91-3.32)	2.46(1.94-2.93)	0.449
eGFR, L/min/1.73m^2 (IQR)	62.2(45.0-79.5)	59.3(47.5-72.5)	0.546
**Stroke location**			
posterior circulation, n%	117(33.1)	21(30.4)	0.672
Insula†, n%	63(18.3)	9(15.3)	0.577
**ICH†, n%**	111(32.2)	15(25.4)	0.301
**sICH†, n%**	91(26.4)	19(32.2)	0.353
**Mortality‡, n%**	94(29.0)	25(39.1)	0.111

hs-cTnI, high-sensitivity cardiac troponin; AF, atrial fibrillation; CAD, coronary artery disease; OTA, onset-to-admission time; IQR, interquartile range; IV rtPA, intravenous recombinant tissue plasminogen activator; NIHSS, National Institutes of Health Stroke Scale score; HbA1c, glycated hemoglobin; HCY, homocysteine; INR, international normalized ratio; LDL-C, low-density lipoprotein; eGFR, estimated glomerular filtration rate; ICH, Intracranial hemorrhage; sICH, symptomatic intracranial hemorrhage.

*control group

† Data were available in 404 patients.

‡data were available in 388 patients

Table S3 Clinical characteristics and mortality of patients, stratified by the presence of Insula involvement

	Insula involvement		*P*
	Yes (n=72)	No (n=332)	
Age >70, n (%)	46(63.9)	189(56.9)	0.278
Male, n (%)	43(59.7)	192(57.8)	0.768
**Vascular risk factors**			
Hypertension, n(%)	38(52.8)	221(66.6)	**0.027**
Diabetes mellitus, n(%)	19(26.4)	93(28.0)	0.780
Current smoking, n(%)	13(18.1)	58(17.5)	0.906

**Comorbidities**			
AF, n(%)	43(59.7)	163(49.1)	0.102
CAD, n(%)	7(9.7)	32(9.6)	0.983
Previous stroke, n(%)	14(19.4)	50(15.1)	0.356

AF, atrial fibrillation; CAD, coronary artery disease;

Table S4.Clinical characteristics and mortality according to hs-cTnI on admission.

	hs-cTnI elevation before MT		*P*
	Yes (n=72)	No (n=351)	
Age >70, n (%)	53(73.6)	193(55.0)	0.004
Male, n (%)	35(48.6)	213(60.7)	0.058
**Vascular risk factors**			
Hypertension, n(%)	42(58.3)	233(66.4)	0.192
Diabetes mellitus, n(%)	21(29.2)	95(27.1)	0.716
Current smoking, n(%)	7(9.7)	68(19.4)	0.051
**Comorbidities**			
AF, n(%)	48(66.7)	164(46.7)	**0.002**
CAD, n(%)	13(18.1)	29(8.3)	**0.011**
Previous stroke, n(%)	12(16.7)	54(15.4)	0.785
**Treatment status**			
Previous antithrombotic therapy, n%	19(26.4)	53(15.1)	**0.020**
OTA, min (IQR)	145(58-275)	105(60-240)	0.564
Transfers, n(%)	9(12.5)	47(13.4)	0.839
Use of IV rtPA, n(%)	14(19.4)	101(28.8)	0.105
Poor reperfusion, n(%)	19(26.4)	65(18.5)	0.127
**NIHSS on admission**			

0-8*, n(%)	7(9.7)	52(14.8)	0.223
9-16, n(%)	22(30.6)	127(36.2)	
>16, n(%)	43(59.7)	172(49.0)	
**Laboratory test**			
hs-cTnI dynamic changes†, n (%)	36(50.0)	54(15.4)	**<0.001**
Baseline blood glucose, mmol/L (IQR)	7.30(6.30-8.20)	7.00(6.10-8.80)	0.643
HbA1c,% (IQR)	6.10(5.55-7.00)	5.90(5.50-6.90)	0.478
HCY, µmol/L (IQR)	15.02(10.23-21.05)	13.30(10.12-18.21)	0.249
Platelet, 10^9/L (IQR)	179(151-228)	194(157-232)	0.327
INR (IQR)	1.06(1.00-1.14)	1.02(0.95-1.09)	**<0.001**
LDL-C mmol/L (IQR)	2.25(1.69-2.82)	2.58(1.97-3.33)	**0.004**
eGFR, L/min/1.73m^2 (IQR)	55.37(41.12-78.21)	63.48(47.27-78.20)	0.055
**Stroke location**			
posterior circulation, n%	20(27.8)	118(33.6)	0.336
Insula‡, n%	18(26.9)	54(16.0)	**0.034**
**ICH**‡	21(31.3)	105(31.2)	**0.976**
**sICH**‡	16(23.9)	94(27.9)	**0.500**
**Mortality****, n%**	29(45.3)	90(27.8)	**0.005**

hs-cTnI, high-sensitivity cardiac troponin; MT, mechanical thrombectomy; AF, atrial fibrillation; CAD, coronary artery disease; OTA, onset-to-admission time; IQR, interquartile range; IV rtPA, intravenous recombinant tissue plasminogen activator; NIHSS, National Institutes of Health Stroke Scale score; HbA1c, glycated hemoglobin; HCY, homocysteine; INR, international normalized ratio; LDL-C, low-density lipoprotein; eGFR, estimated glomerular filtration rate; END, early neurological deterioration; ICH, Intracranial hemorrhage; sICH, symptomatic intracranial hemorrhage.

*control group

† Data were available in 354 patients.

‡data were available in 404 patients

**data were available in 388 patients

Table S5.Kaplan-Meier survival analysis and multivariate cox regression analysis of the predictors of mortality.

	**Univariate model**		**Multivariable model 1**		**Multivariable model 2**	
	**HR(95% CI)**	**p**	**aHR(95% CI)**	**p**	**aHR(95% CI)**	**p**
Age>70, n (%)	1.892(1.314-2.725)	**<0.001**				
Male, n (%)	1.087(0.753-1.569)	0.657				
**Vascular risk factors**						
Hypertension, n(%)	1.607(1.104-2.338)	**0.013**	1.645(1.035-2.615)	0.035	1.747(1.066-2.862)	0.027
Diabetes mellitus, n(%)	1.190(0.794-1.784)	0.399				
Current smoking, n(%)	0.949(0.590-1.527)	0.829				
**Comorbidities**						
AF, n(%)	1.064(0.741-1.529)	0.736				
CAD, n(%)	1.158(0.627-2.137)	0.639				
Previous stroke, n(%)	1.030(0.625-1.697)	0.908				
**Treatment status**						
Previous antithrombotic therapy, n%		0.700				
OTA>270 min, n(%)	0.915(0.579-1.445)	0.703				
Transfers, n(%)	1.194(0.698-2.040)	0.518				
Use of IV rtPA, n(%)	0.558(0.373-0.833)	**0.004**	0.426(0.236-0.772)	0.005	0.427(0.231-0.791)	0.007
Poor reperfusion, n(%)	5.038(3.081-8.239)	**<0.001**	2.881(1.865-4.452)	<0.001	2.719(1.706-4.336)	<0.001
Dual antiplatelet therapy, n(%)	0.452(0.265-0.774)	**0.004**				
**NIHSS on admission**		**<0.001**		<0.05		
0-8*, n(%)						

9-16, n(%)	2.979(1.045-8.494)	**0.041**	2.230(0.771-6.453)	0.139	3.459(1.031-11.610)	0.045
>16, n(%)	7.571(2.778-20.634)	**<0.001**	4.692(1.704-12.918)	0.003	8.052(4.409-14.705)	0.001
**Laboratory test**						
hs-cTnI elevation on admission, n(%)	1.907(1.162-3.131)	**0.011**				
hs-cTnI dynamic changes, n (%)	2.618(1.610-4.256)	**<0.001**	1.613(1.056-2.463)	0.027	1.597(1.022-2.495)	0.040
Baseline blood glucose>7mmol/L , n (%)	1.616(1.113-2.349)	**0.012**				
Platelet, n (%)		0.175				
<100*10^9/L[*]						
100-300*10^9/L						
>300*10^9/L						
INR, n%		0.545				
<0.82[*]						
0.82-1.15						
>1.15						
LDL-C >1.8 mmol/L, n%	0.936(0.563-1.557)	0.800				
eGFR<60L/min/1.73m^2, n%	1.485(1.014-2.176)	**0.042**				
**Stroke location**						
posterior circulation, n%	2.044(1.383-3.023)	**<0.001**	1.577(1.044-2.384)	0.030		
Insula lesion, n%		0.204				
sICH, n%	3.531(2.219-5.620)	**<0.001**			2.259(1.447-3.529)	<0.001

Model 1: adjusted for the age>70, hypertension, use of IV rtPA, dual antiplatelet therapy, Poor reperfusion, NIHSS on admission, baseline blood glucose>7mmol/L, eGFR<60L/min/1.73m2, and posterior circulation.

Model 2: adjusted for the age>70, Hypertension, use of IV rtPA, dual antiplatelet therapy,, Poor reperfusion, NIHSS on admission, Baseline blood glucose>7mmol/L, eGFR<60L/min/1.73m2, posterior circulation, sICH.

HR, hazard ratio; CI, confidence interval; aHR, adjusted hazard ratio; AF, atrial fibrillation; CAD, coronary artery disease; OTA, onset-to-admission time; IV rtPA, intravenous recombinant tissue plasminogen activator; NIHSS, National Institutes of Health Stroke Scale score; hs-cTnI, high-sensitivity cardiac troponin; INR, international normalized ratio; LDL-C, low-density lipoprotein; eGFR, estimated glomerular filtration rate; sICH, symptomatic intracranial hemorrhage.
*control group

Table S6. Comparison of Model 1among original data and multiple imputation for multivariate cox regression analysis.

	**Original data**			**Multiple Imputation**		
	**aHR**	**95% CI**	**p**	**aHR**	**95% CI**	**p**
Hypertension, n(%)	1.805	1.074-3.034	0.026	1.645	1.035-2.615	0.035
Use of IV rtPA, n(%)	0.476	0.256-0.886	0.019	0.426	0.236-0.772	0.005
Poor reperfusion, n(%)	2.444	1.505-3.968	<0.001	2.881	1.865-4.452	<0.001
NIHSS on admission			<0.05			<0.05
0-8*, n(%)						
9-16, n(%)	1.952	0.664-5.735	0.224	2.230	0.771-6.453	0.139
>16, n(%)	4.006	1.440-11.146	0.008	4.692	1.704-12.918	0.003
hs-cTnI dynamic changes, n (%)	1.702	1.082-2.679	0.021	1.613	1.056-2.463	0.027
posterior circulation, n%	1.585	1.014-2.478	0.043	1.577	1.044-2.384	0.030

aHR, adjusted hazard ratio; CI, confidence interval; IV rtPA, use of intravenous recombinant tissue plasminogen activator; NIHSS, National Institutes of Health Stroke Scale score; hs-cTnI, high-sensitivity cardiac troponin.
*control group

Table S7. Comparison of Model 2 among original data and multiple imputation for multivariate cox regression analysis.

	Original data			Multiple Imputation		
	**aHR**	**95% CI**	**p**	**aHR**	**95% CI**	**p**
Hypertension, n(%)	1.800	1.052-3.081	0.032	1.747	1.066-2.862	0.027
Use of IV rtPA, n(%)	0.452	0.237-0.861	0.016	0.427	0.231-0.791	0.007
Poor reperfusion, n(%)	2.533	1.523-4.213	<0.001	2.719	1.706-4.336	<0.001
NIHSS on admission			<0.001			<0.05
0-8*, n(%)						
9-16, n(%)	3.210	0.940-10.955	0.063	3.459	1.031-11.610	0.045
>16, n(%)	7.901	2.394-26.081	0.001	8.052	4.409-14.705	0.001
hs-cTnI dynamic changes, n (%)	1.753	1.097-2.800	0.019	1.597	1.022-2.495	0.040
sICH, n%	2.621	1.625-4.229	<0.001	2.259	1.447-3.529	<0.001

aHR, adjusted hazard ratio; CI, confidence interval; IV rtPA, use of intravenous recombinant tissue plasminogen activator; NIHSS, National Institutes of Health Stroke Scale score; hs-cTnI, high-sensitivity cardiac troponin; sICH, symptomatic intracranial hemorrhage.

*control group

Figure.S3 Differences in 90-day mortality between groups with non-dynamic, rising and falling dynamic changes.

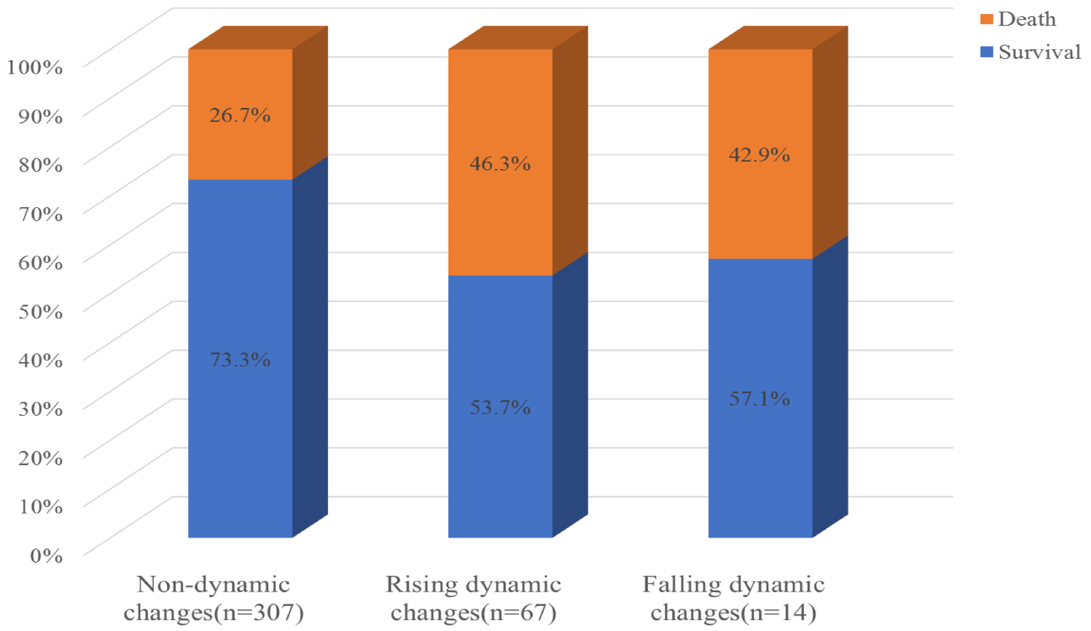

Notes: The proportions of lost to following-up were 26/333(7.8%) in Non-dynamic changes, 8/75(10.7%) in Rising dynamic changes, 1/15(6.7%) in Falling dynamic changes, respectively.

Table S8. Subgroup analysis to identify relationships between hs-cTnI dynamic changes and mortality

	aHR	95% CI	*P*	*P* for interaction
Age, years				0.023
≤ 70			>0.05	
> 70			>0.05	
Sex, n%				>0.05
Female	2.833	1.338-5.996	**0.007**	
Male			>0.05	
AF, n%				>0.05
No	2.041	1.028-4.053	0.042	
Yes			>0.05	
CAD, n%				>0.05
No			>0.05	
Yes			>0.05	
NIHSS score				>0.05

≤ 8*			>0.05	
9-16			>0.05	
>16			>0.05	
Insula lesion, n%				>0.05
No	1.801	1.119-2.898	**0.015**	
Yes			>0.05	
posterior circulation, n%				>0.05
No	2.850	1.522-5.336	**0.001**	
Yes			>0.05	
sICH, n%				>0.05
No	2.631	1.433-4.831	**0.002**	
Yes			>0.05	
Dual antiplatelet therapy, n%				>0.05
No			>0.05	
Yes			>0.05	

hs-cTnI, high-sensitivity cardiac troponin; aHR, adjusted hazard ratio; CI, confidence interval; AF, atrial fibrillation; CAD, coronary artery disease; NIHSS, National

BMJ Publishing Group Limited (BMJ) disclaims all liability and responsibility arising from any reliance placed on this supplemental material which has been supplied by the author(s)

Institutes of Health Stroke Scale score; sICH, symptomatic intracranial hemorrhage.

*control group
